# A Prospective Study of Vitamin D Supplement in Thyroidectomy Patients Based on Relative Decline of Parathyroid Hormone

**DOI:** 10.3389/fphar.2021.626614

**Published:** 2021-03-08

**Authors:** Qing Hao, Yun Qin, Wanjun Zhao, Lingyun Zhang, Han Luo

**Affiliations:** ^1^ Department of Thyroid and Parathyroid Surgery, Laboratory of thyroid and parathyroid disease, Frontiers Science Center for Disease-related Molecular Network, State Key Laboratory of Biotherapy and Cancer Center, West China Hospital, Sichuan University, Chengdu, China; ^2^ Department of Radiology, West China Hospital, Sichuan University, Chengdu, China

**Keywords:** thyroidectomy, vitamin D, parathyroid hormone, prospective study, hypocalcemia

## Abstract

**Background:** In postthyroidectomy patients, hypocalcemia is the most common complication to prolong hospital stay and decrease patients’ satisfaction. Based on current evidence, it is recommended to supply vitamin D to patients with high risk of developing hypocalcemia. However, how to stratify the risk of patients remains challenging.

**Aim:** We conducted a prospective study to evaluate the effect of vitamin D supplement (calcitriol) on high-risk hypocalcemia patients based on relative decline of parathyroid hormone (RDP).

**Method:** RDP was calculated by the difference between preoperative and postoperative first-day PTH divided by preoperative PTH and presented as percentage. Patients who underwent total thyroidectomy in addition to bilateral central compartment dissection were enrolled prospectively and were divided into two cohorts: Cohort I: patients with RDP ≤70% and Cohort II: patients with RDP >70%. Patients in Cohort I were then randomly assigned to Group A or B, and patients in Cohort II were randomly assigned to Group C or D. All groups received oral calcium, and patients in Groups B and D also received calcitriol. All patients were followed for one year. In the study, standard procedure dictates that only oral calcium is given to patients whose RDP ≤70% and that oral calcium and calcitriol are given to patients whose RDP >70%. Therefore, Cohort I Group A and Cohort II Group D are controls in this study.

**Results:** The incidence of clinical hypocalcemia in Groups A and D (the controls) was 11.0% (10/91), and 17.6% (16/91) required additional intravenous calcium. Of note, no patients developed permanent hypocalcemia. Furthermore, calcitriol supplement did not have significant impact on clinical outcomes between Group A and B in Cohort I. By contrast, calcitriol supplement distinctly improved clinical outcome by comparing Groups C and D (Cohort II), as marked by clinical hypocalcemia, need of requiring intravenous calcium, and long-termed decreased levels of PTH.

**Conclusion:** Supplying calcitriol based on RDP cutoff of 70% may be a wise practice in thyroidectomy patients, and RDP 70% may be a useful predictor to stratify high-risk patients.

## Background

Hypocalcemia is the most common complication of thyroidectomy with incidence of biochemical hypocalcemia reported to be 22%–83% (Wingert et al., 1986; Noordzij et al., 2007; Mehanna et al., 2010; Sheahan et al., 2013) and clinical hypocalcemia 10% to 50% (Noordzij et al., 2007; Sheahan et al., 2013). Here, biochemical hypocalcemia was defined as the measured serum calcium level less than 2.1 mmol/L (8.4 mg/dl). Patients with clinical hypocalcemia typically exhibit perioral or fingertip numbness with or without Chvostek sign on top of biochemical hypocalcemia. If hypocalcemia lasts longer than 6 months after surgery, it is considered permanent or persistent hypocalcemia and has an incidence of 2–8% (Jacobs et al., 1983; Reeve and Thompson, 2000; Mehanna et al., 2010). Persistent clinical hypocalcemia is the main factor to prolong hospital stay and decrease patients’ subjective satisfaction (Erbil et al., 2007).

It is well established that the postoperative calcium level predicts symptomatic hypocalcemia after surgery, and the calcium level is regulated by vitamin D and parathyroid hormone (Peacock, 2010). Vitamin D_3_ hydroxylates into the active form (1, 25-dihydroxyvitamin D_3_, also known as calcitriol) via hydroxylase in the liver and kidney (Erbil et al., 2007; Peacock, 2010). Physiologically, calcitriol is an important regulator for calcium absorption in intestine. Testa et al. (2006) proved vitamin D supplement could decrease hypocalcemia symptoms significantly. Moreover, Roh and Park (2006) also demonstrated that calcium in combination with vitamin D significantly decreased hypocalcemia symptoms from 36.7% to 8.2%. However, Nhan et al. (2012) showed that preoperative low vitamin D (defined as 25(OH)D ≤ 70 nmol/L) was not a predictor of clinical hypocalcemia. Similarly, Bellantone et al. (2002), Tartaglia et al. (2005), and Pisaniello et al. (2005) found that calcium supplemented by vitamin D did not have a significant impact on clinical hypocalcemia compared with calcium alone. Therefore, the data on the effects of calcitriol on reducing postoperative clinical hypocalcemia continue to be inconclusive. In considering these data, Alhefdhi et al. (2013) recommended oral calcium for all patients following thyroidectomy, with the addition of vitamin D supplement for high-risk individuals.

However, how to stratify thyroidectomy patients with high risk remains a challenge. Though multiple studies showed that the absolute value of postoperative serum parathyroid hormone (PTH) was an accurate predictor of high-risk hypocalcemia in thyroidectomy patients, cutoff value was variant (Edafe et al., 2014). On the other hand, relative parathyroid decline was proved to be a reliable predictor. In our previous preliminary study (Luo et al., 2017), we found that relative 70% decline of PTH was a reasonable indicator of clinical hypocalcemia at 4.67% vs. 27.0% in patients with relative decline of ≤70% and >70%, respectively. Therefore, we hypothesized that PTH relative decline (RDP) may contribute to identifying high-risk patients of hypocalcemia who need calcitriol supplement after thyroidectomy.

Here, we conducted a prospective study of calcitriol supplement based on RDP to evaluate the effect of calcitriol supplement in thyroidectomy patients.

## Materials and Methods

This prospective, randomized, parallel-arm study was conducted between July and December of 2016. The trail was registered in *Chinese Clinical Trial Registry* with number: *ChiCTR-IOR-16008929*. The protocol was approved by the ethical committee of West China Hospital. Written informed consent was obtained from each patient.

All patients included in this study met the following criteria: (1) diagnosis of papillary thyroid carcinoma (PTC) by fine needle aspiration (FNA); (2) treatment by total thyroidectomy and bilateral central compartment nodal dissection (CND); (3) age between 18 and 80 years; and (4) ability to understand and consent to study protocol. During the surgery, the parathyroid glands were carefully preserved, and the devascularized parathyroid glands were transplanted into the sternocleidomastoid muscle. Autotransplantation of the parathyroid glands was not performed routinely.

For all patients, the PTH level was determined by electrochemiluminescence immunoassay (Cobas, Roche Diagnostics, Mannheim, Germany). The serum calcium level was measured using Cobas 8000 modular biochemistry analyzer (Roche Diagnostics GmbH, Mannheim, Germany). Postoperative first-day (POD1) PTH was measured in the early morning of first day after surgery. Relative decline of PTH (RDP) was calculated in all patients according to the formula [(PTH _p.o._ − POD1 PTH)/PTH _p.o._] × 100%, where PTH _p.o._ referred to preoperative PTH. Reference range for the normal value of PTH is 1.6–6.9 pmol/L and for calcium is 2.1–2.7 mmol/L.

**FIGURE 1:**
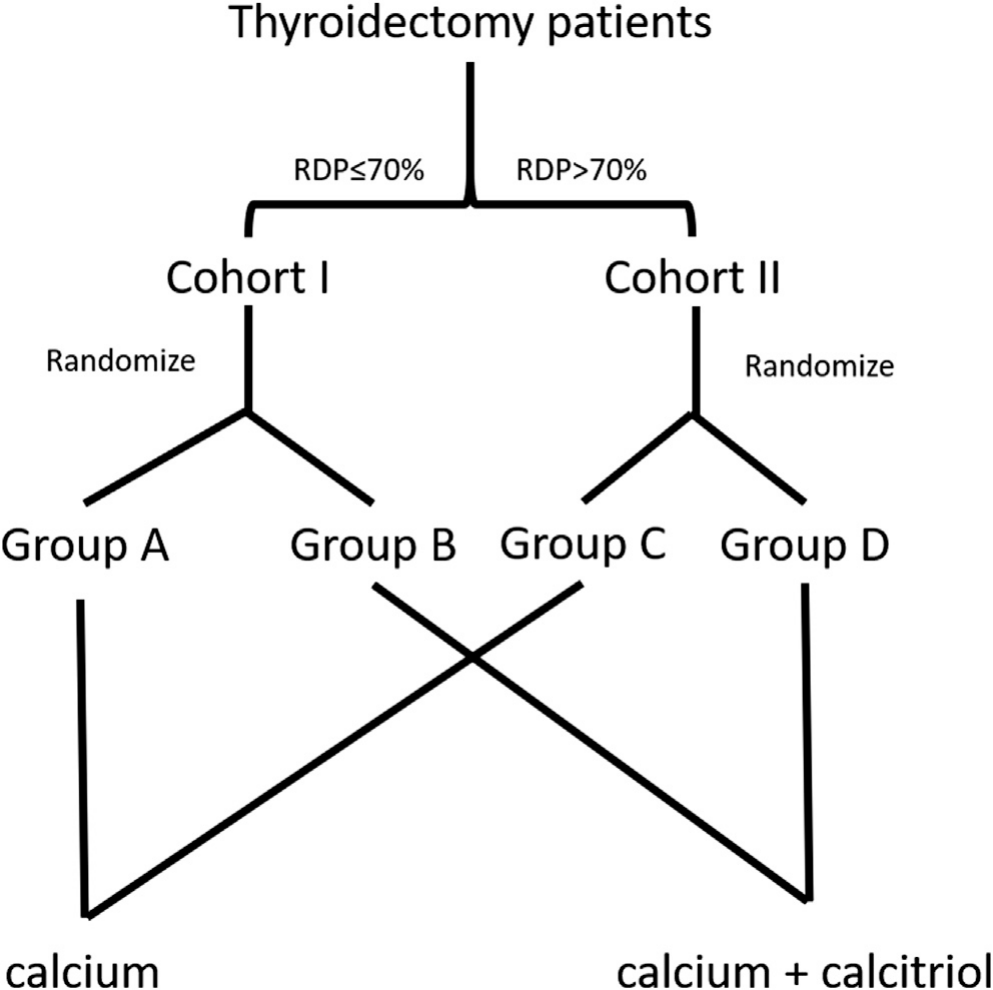
The flow diagram of enrollment and randomization in the randomized clinical trial. Patients were divided in two cohorts based on relative decline of parathyroid hormone (RDP)—Cohort I ≤70% and Cohort II >70%. Subsequently, patients in either cohort were allocated randomly into two groups, respectively. The standard procedure is done in Group A and Group D.

Patients were divided into two cohorts according to RDP (Cohort I: ≤70% and Cohort II: >70%). Then, patients in Cohort I were allocated into Group A or B randomly. Likewise, patients in Cohort II were allocated into Group C or D randomly. All postoperative patients in the four groups were prescribed oral calcium carbonate 600 mg t.i.d for 3 days. In addition to calcium, Group B and D patients were prescribed calcitriol 0.25 μg b.i.d. for 3 days. The pipeline is shown in Figure 1. In the study, the standard procedure is to give calcitriol to patients whose RDP >70% and no calcitriol to patients whose RDP <70%. Therefore, Cohort I Group A and Cohort II Group D are controls in this study.

Hypocalcemia symptoms (perioral or fingertip numbness, Chvostek sign, and persistent time) and need for intravenous (i.v.) calcium were evaluated independently by nurse and physician. The need for i.v. calcium was mainly decided by patients’ hypocalcemia symptoms regardless of the calcium level. All patients were followed for one year with follow-up at the first month, third month, sixth month, and twelfth month after surgery.

In this RCT protocol, in order to fully and better assess the efficacy of our standard procedure on hypocalcemia symptoms management, we ruled that all patients will be discharged in 4–5 days after thyroidectomy because, based on the previous retrospective report, majority of hypocalcemia patients will show symptoms in 1–2 days after surgery.

Sample size was calculated based on our preliminary study using Power Analysis and Sample Size version 15.0 (PASS 15.0, NCSS, LLC, Kaysville, UT, United States). The preliminary results showed that clinical hypocalcemia occurred in 27.0% of patients with RDP >70%. We expected that there would be at least 17% decline in the incidence of clinical hypocalcemia under our standard procedure (Group D). To achieve a statistical power of 80% and a 2-sided type I error of 5%, 44 patients were needed in each group. With an assumption of an approximate 8% dropout rate, our aim was to enroll 48 patients in each group of Groups C and D.

Given that clinical hypocalcemia occurred in only 4.67% of patients with RDP ≤70% in our preliminary study, and the aim of our present study was mainly to assess the efficacy of the standard procedure on preventing hypocalcemia symptoms in patients with RDP >70%, and we set the sample size of Group I (Group A and Group B) same as Group II.

Data analysis was performed using SPSS version 21 (SPSS Inc., Chicago, IL, United States). If normally distributed, continuous variables were presented as mean ± standard deviation and compared using Student’s *t*-test. The paired *t*-test was also used when needed; otherwise, variables were presented as median (range) and compared using the Mann–Whitney *U* test. Pearson’s chi-square test or Fisher’s exact test was used to compare the frequency (percentage) of categorical variables. The *p* value<0.05 indicated significant difference.

## Result

A total of 182 thyroidectomy patients were enrolled into the prospective study, where Cohort I consisted of 88 patients with 44 in both Groups A and B and Cohort II included 94 patients with 47 in both Groups C and D. Baseline comparison of all groups showed no significant difference in all variables other than body mass index (BMI) and PTH_p.o_ in Table 1. Though baseline of PTH _p.o._ was incomparable, no enrolled patients had aberrant PTH level before surgery.

**TABLE 1:** Baseline characteristic comparison of four Groups.

	Group A	Group B	Group C	Group D	*p* value
	N = 44	N = 44	N = 47	N = 47	
Age	41.49 ± 11.36	41.58 ± 10.24	41.83 ± 9.85	41.49 ± 11.36	0.998
Gender (male/female)	11/33	14/30	18/29	13/34	0.668
BMI	22.96 ± 3.39	22.35 ± 3.63	24.50 ± 4.53	23.04 ± 3.27	0.046
Tumor size	9.27 ± 3.97	10.47 ± 4.36	10.39 ± 5.59	10.73 ± 9.45	0.702
Isthmus located (Yes/No)	7/37	3/41	9/38	8/39	0.518
ETE (Yes/No)	20/27	21/23	24/23	28/19	0.103
Operation duration	2.36 ± 0.51	2.51 ± 0.76	2.38 ± 0.60	2.56 ± 0.62	0.347
Intraoperative fluid volume	1174.63 ± 425.10	1131.40 ± 362.04	1252.17 ± 495.64	1198.08 ± 356.07	0.581
TNM Stage					
T (T1-2/T3-4)	32/12	29/15	31/16	29/18	0.327
N (N0/N1)	36/8	35/9	41/6	34/13	0.456
M	0	0	0	0	
Fluid volume in first 24 h	1577.61 ± 357.95	1569.77 ± 351.55	1515.22 ± 418.98	1483.54 ± 393.03	0.597
Drainage in first 24 h	53.46 ± 28.58	52.26 ± 20.27	52.65 ± 26.55	58.52 ± 23.71	0.597
Total drainage	77.69 ± 45.62	83.67 ± 36.59	89.13 ± 45.11	93.13 ± 38.75	0.321
Preoperative calcium	2.31 ± 0.11	2.35 ± 0.09	2.31 ± 0.12	2.29 ± 1.00	0.956
Preoperative magnesium	0.88 ± 0.06	0.88 ± 0.07	0.87 ± 0.12	0.89 ± 0.06	0.706
Preoperative PTH	5.99 ± 2.20	5.33 ± 1.95	7.78 ± 6.67	6.11 ± 1.98	0.018
Preoperative albumin	46.08 ± 8.27	46.15 ± 7.10	47.32 ± 3.22	46.91 ± 3.14	0.692
TSH	2.98 ± 1.65	3.27 ± 2.35	3.41 ± 2.27	3.44 ± 1.97	0.711
Postoperative hospital stay	4.42 ± 1.93	4.79 ± 1.42	4.85 ± 1.52	5.06 ± 1.51	0.294

**FIGURE 2:**
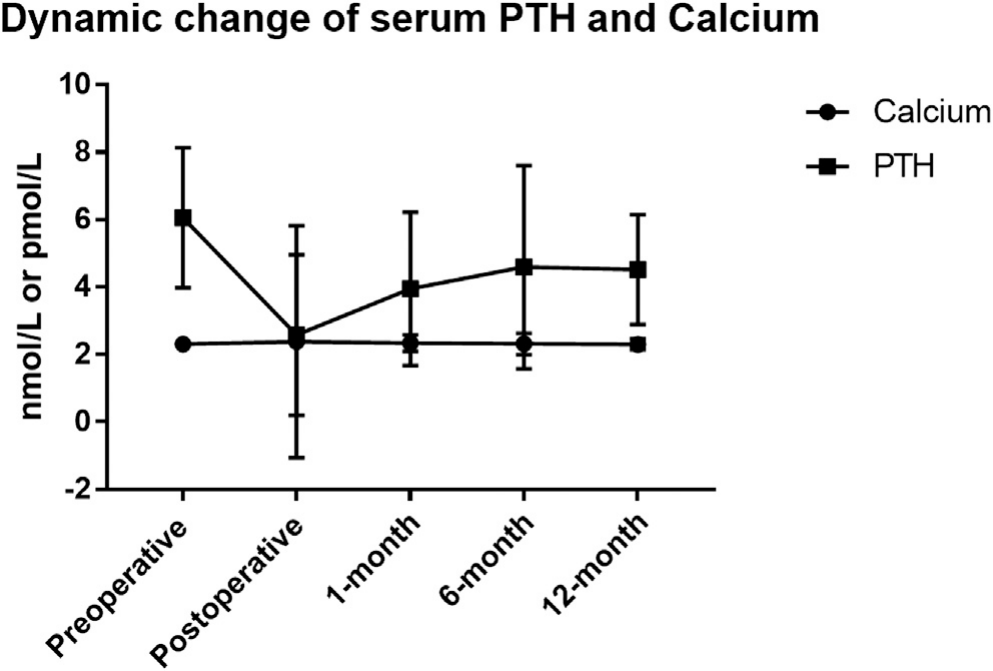
Serum parathyroid hormone (PTH) and calcium dynamic change in thyroidectomy patients of standard pipeline. The standard procedure is done in Group A and Group D. Patients in Group A were prescribed oral calcium carbonate 600 mg t.i.d for 3 days. In addition to calcium, Group D patients were prescribed calcitriol 0.25 μg b.i.d. for 3 days.

Firstly, the total incidence of clinical hypocalcemia in standard procedure (Groups A and D) was 11.0% (10/91), and 17.6% (16/91) patients required i.v. calcium. The dynamic change of serum calcium and PTH from preoperative to postoperative 1 year is shown in Figure 2. No patient developed permanent hypocalcemia or hypoparathyroidism. The details of Groups A and D are presented in Table 2.

**TABLE 2:** Efficacy comparison of calcitriol supplement.

	Group A	Group B	*p* value	Group C	Group D	*p* value
	N = 44	N = 44		N = 47	N = 47	
Symptomatic hypocalcemia (Yes/No)	4/40	3/41	>0.999	26/21	6/41	<0.001
Numbness	3	4	—	24	4	—
Twitch	2	0	—	3	2	—
Chvostek’s sign	0	0	—	1	1	—
Location of symptom			>0.999			0.005
Perioral	0	0	—	2	2	—
Finger and toe	0	0	—	6	2	—
Facial	0	0	—	2	1	—
Limb	4	4	—	20	3	—
Extent of numbness			>0.999			<0.001
Mild	4	4	—	24	4	—
Moderate	0	0	—	2	1	—
Severe	0	0	—	0	1	—
Persistent symptom more than 5 min	1	1	>0.999	12	0	<0.001
1st month PTH	4.88 ± 1.75	4.84 ± 1.60	0.911	3.66 ± 1.56	4.21 ± 1.65	0.100
1st month calcium	2.30 ± 0.13	2.31 ± 0.14	0.729	2.33 ± 0.54	2.25 ± 0.14	0.328
6th month PTH	5.65 ± 2.71	5.46 ± 2.21	0.719	3.77 ± 1.16	4.46 ± 1.69	0.023
6th month calcium	2.39 ± 0.36	2.33 ± 0.13	0.301	2.25 ± 0.25	2.31 ± 0.49	0.465
1st year PTH	6.02 ± 3.00	5.07 ± 1.54	0.065	3.98 ± 1.40	5.13 ± 2.30	0.043
1st year calcium	2.36 ± 0.09	2.49 ± 0.55	0.126	2.26 ± 0.14	2.28 ± 0.16	0.521
i.v. calcium required	8	8	>0.999	22	8	0.004

i.v.: intravenous

**FIGURE 3:**
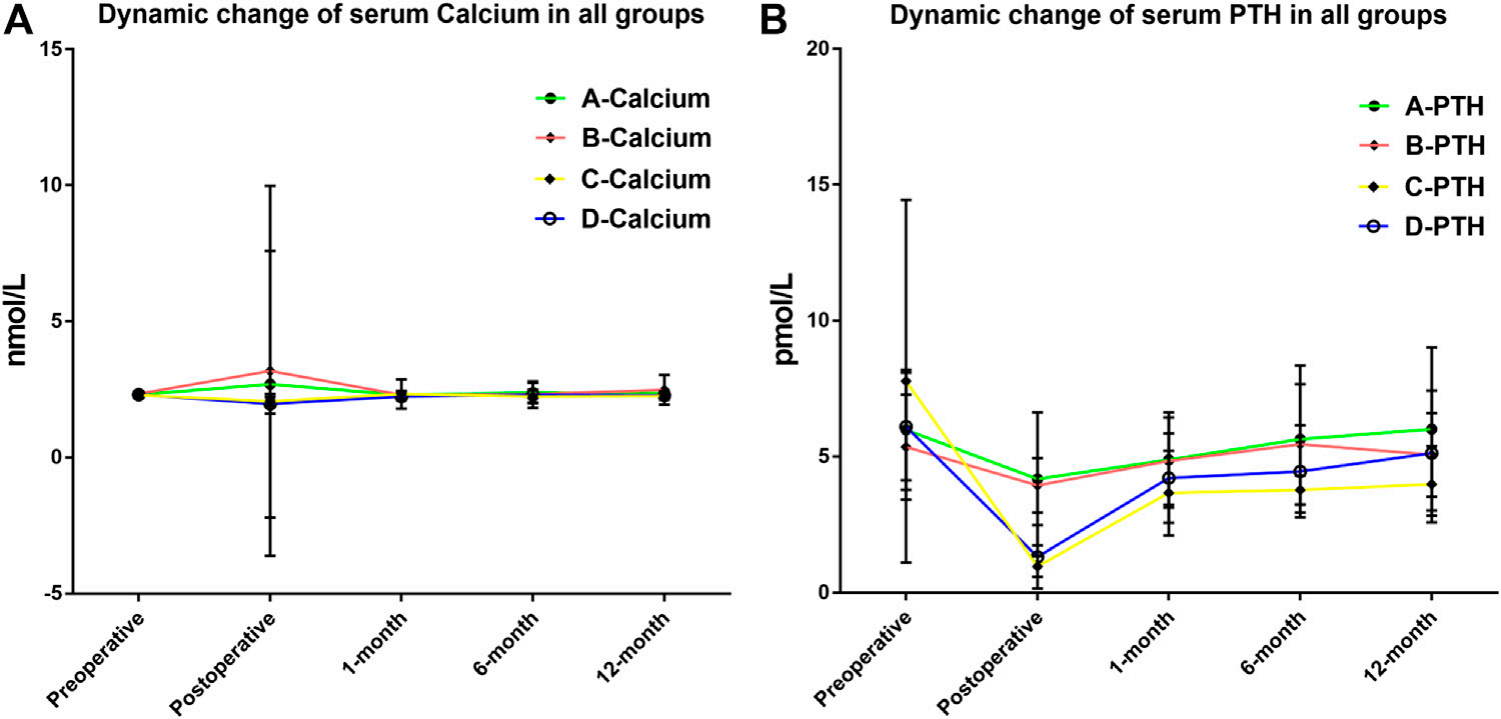
**(A)** Dynamic fluctuation of serum calcium after thyroidectomy in all four groups. No significant difference was found at any time point during the follow-up. **(B)** Dynamic fluctuation of serum parathyroid hormone (PTH) after thyroidectomy in four groups. Postoperative PTH level in Group D was significantly higher than that in Group C (1.32 ± 1.16 vs. 0.96 ± 0.38 pmol/L, *p* = 0.024). At 12-month evaluation, PTH was significantly higher in Group D than that in Group C (5.13 ± 2.30 vs. 3.98 ± 1.40 pmol/L, *p* = 0.020).

Then, Groups B and C were compared with their respective control group (A and D, respectively) to evaluate the efficiency of RDP-based postoperative management procedure. The data clearly demonstrated that prescription with or without calcitriol in patients with RDP ≤70% did not have significant impact on outcome (Groups A and B) in Table 2. However, clinical outcome was distinctly different between Groups C and D, namely, in clinical hypocalcemia, i.v. calcium requirement, and long-term PTH level at 6 and 12 months after surgery. This suggests that calcitriol supplement in patients with RDP >70% is associated with fewer incidences of clinical hypocalcemia and patients’ requirement for i.v. calcium, as well as elevated PTH level. The dynamic changes of calcium and PTH in Groups A/B/C/D are displayed in Figure 3. The postoperative PTH level in Group D was significantly higher than that in Group C (1.32 ± 1.16 vs. 0.96 ± 0.38 pmol/L, *p* = 0.024), but no difference was detected between Group A and B (*p* = 0.174). At 12-month evaluation, PTH was significantly higher in Group D than that in Group C (5.13 ± 2.30 vs. 3.98 ± 1.40 pmol/L, *p* = 0.020). And no patient in Group A/B/D was still prescribed calcitriol or calcium. However, still there were four patients in Group C (without calcitriol) at 12-month evaluation. We have added this information in the result part.

## Discussion

This is a prospective study to evaluate effect of calcitriol supplement in postthyroidectomy patients based on PTH change. In our previous research, 70% relative decline of PTH (RDP) was selected as a cutoff to predict clinical hypocalcemia. In the present study, 11.0% (10/91) patients developed clinical hypocalcemia in standard procedure, which was lower than the British Association of Endocrine and Thyroid Surgeons (2016) audit reported ratio of 27.4% (Ferris et al., 2015). Both Docimo et al. (2012) and Maxwell et al. (2017) reported 6% clinical hypocalcemia occurred in their research studies; however, neither performed routine central compartment dissection. Therefore, we believe RDP-based management may be effective in reducing clinical hypocalcemia.

In the present study, it was demonstrated that 70% was an effective indicator to stratify patients with high risk of clinical hypocalcemia who need calcitriol after surgery as 12.8% (6/47) and 55.3% (26/47) developed clinical hypocalcemia in Groups C and D, respectively. Therefore, it indicated the necessity of calcitriol supplement for patients with RDP >70%. Conversely, it may be unnecessary to supply calcitriol for patients with RDP ≤70% as demonstrated by similar incidences of hypocalcemia at 9.10% (4/44) and 6.82% (3/44) in Groups A and B, respectively, with *p* value >0.999.

Limited by the sample size of the study, though it was a prospective study, the results still need to be validated in a larger cohort or clinical practice. Additionally, the preoperative baseline level of vitamin D was not taken into consideration because whether vitamin D deficiency is a risk factor of hypocalcemia is controversial (Kim et al., 2015; Carvalho et al., 2019; Manzini et al., 2019). Besides, multivariate analysis was not done due to the small sample size and low event incidence; thus, the effect of several confounding factors including the gender, age, and diet on the serum calcium level of patients could not be well dissected. Furthermore, the baseline characteristics of the four groups of patients were not wholly comparable (Table 1). Previously, Edafe et al. proved that higher preoperative PTH was not a risk factor of hypocalcemia in a systematic review (Edafe et al., 2014), while Al-Dhahri et al. (2014) found that patients’ BMI and preoperative PTH level may affect the speed of recovery of hypoparathyroidism.

In conclusion, RDP-based postoperative management may be effective in preventing clinical hypocalcemia in thyroidectomy patients. In terms of patients with RDP more than 70%, calcitriol supplement is necessary; however, for patients with RDP lower than 70%, calcitriol is unnecessary.

## Data Availability

The raw data supporting the conclusions of this article will be made available by the authors, without undue reservation.
